# A novel elastin gene frameshift mutation in a Russian family with cutis laxa: a case report

**DOI:** 10.1186/s12895-019-0084-6

**Published:** 2019-01-31

**Authors:** E. G. Okuneva, A. A. Kozina, N. V. Baryshnikova, A. Yu Krasnenko, K. Yu Tsukanov, O. I. Klimchuk, E. I. Surkova, V. V. Ilinsky

**Affiliations:** 1 Genotek Ltd., Nastavnicheskii pereulok 17/1, 105120 Moscow, Russia; 2 Institute of Biomedical Chemistry, Pogodinskaya street 10 building 8, 119121 Moscow, Russia; 3 Pirogov Russian National Research Medical University, Ostrovitianova street 1, 117997 Moscow, Russia; 4 Vavilov Institute of General Genetics, Gubkina street 3, 119333 Moscow, Russia

**Keywords:** Cutis laxa, Elastolysis, Elastin, *ELN*, Progeria

## Abstract

**Background:**

Cutis laxa (CL) is a rare connective tissue disorder characterized by loose, redundant, inelastic and wrinkled skin. Patients develop a prematurely aged appearance. Inheritance can be autosomal dominant or autosomal recessive. The X-linked form is now classified in the group of copper transport diseases. Autosomal dominant CL is characterized by wrinkled, redundant and sagging, inelastic skin and in some cases is associated with internal organ involvement.

**Case presentation:**

We report a familial case of autosomal dominant CL, which includes a 33-year-old woman and her 11-year-old son with dry, thin and wrinkled skin that appeared prematurely aged. No serious involvement of internal organs was found. In both patients, we identified novel heterozygous mutation c.2323delG (p.Ala775fs) in exon 34 of elastin transcript NM_001278939.1. Similar frameshift mutations in the last exons of elastin gene were previously reported in patients with autosomal dominant CL.

**Conclusions:**

Our results show a novel frameshift mutation that was found in patients with cutis laxa. Exome sequencing is effective and useful technology for properly diagnosis of diseases with similar phenotype to ensure proper treatment is provided.

## Background

Cutis laxa (CL), or elastolysis, is a group of rare connective tissue disorders characterized by loose, redundant, wrinkled skin [1]. The disease can be inherited or acquired. Inherited form can be autosomal dominant or autosomal recessive. The formerly classified X-linked form, caused by mutations in the *ATP7A* gene, is now classified within the group of copper deficiency syndromes and has been shown to be allelic with Menkes syndrome [2].

CL is caused by mutations in multiple genes, crucial for genesis of elastic fibers [3]. Elastic fibers are extracellular matrix structures responsible for properties of resilience and elastic recoil in all elastic tissues including lungs, large blood vessels and dermis. Histologically, cutis laxa elastic fibers are sparse and fragmented [4].

Differences in clinical presentation of congenital forms of CL depend on the pattern of inheritance. Autosomal dominant cutis laxa (ADCL) is characterized by a generalized loose skin folds and distinctive facial features including long philtrum, a high and broad forehead, prominent ears, and a beak-like nose. Other manifestations occasionally include aortic root dilatation, pulmonary artery stenosis, emphysema and sudden death. ADCL is commonly considered as a milder form of CL with minor systemic involvement and normal neuromotor development whereas ARCL (autosomal recessive form) is more severe with many involved systems and poor prognosis [5].

Elastin gene mutations predominate as the cause of autosomal dominant cutis laxa (OMIM 123700). ELN gene is a single-copy gene with 34 exons localized in humans at 7q11.2. Most patients with ADCL have heterozygous frameshift deletions that are localized in last 5 exons of *ELN* [6–9]. Also, a mutation in exon 25 of *ELN* has been described for ADCL patients [10]. Other ADCL-causing mutations were also identified in *FBLN5* [11] and *ALDH18A1* [12].

In this study we analyzed clinical and genetic characteristics of 33-year-old female proband and her 11-year-old son with dry and wrinkled skin. Differential diagnosis was conducted between progeria and cutis laxa.

## Case presentation

**Fig. 1:**
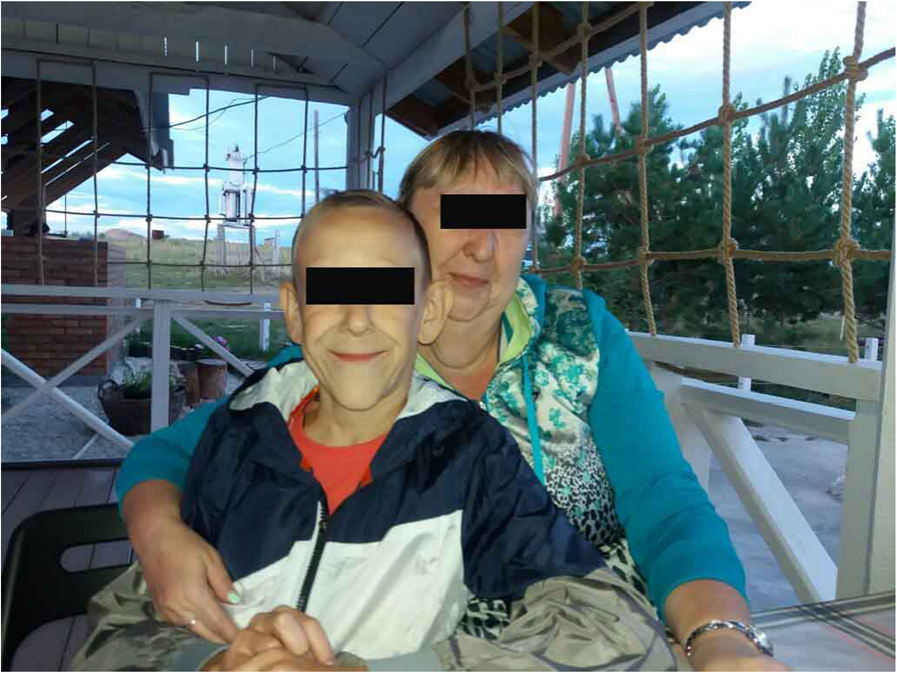
Clinical photograph of proband and her son, ages 33 and 11, respectively

The two patients are the proband and her son from Kazakhstan who are Russian by nationality. Both patients have dry, wrinkled skin and look older than their age (Fig. 1). Family history was negative for similar clinical conditions, indicating de novo mutation occurring in the proband or incomplete penetrance in previous generations.

### Patient 1 mother

Cutis laxa was noted immediately at birth. Height and weight at birth were normal. In childhood, frequent colds were observed. Over the past twenty years, the severity of skin changes has increased (skin has become drier, thinner, and more wrinkled). Weakness and fatigue also progressed.

At the age of 33 years her height and weight were 167 cm (72 percentile) and 49 kg (11 percentile), respectively. Blood pressure was 110/70 mmHg. Skin was dry, thinned and wrinkled. Subcutaneous fat layer was thinned out. Low muscle tone was observed. Hair, nails and teeth were normal. Biochemical blood test did not reveal abnormalities. Respiratory examination revealed normal vesicular breathing, carried out in all lung compartments, there was no wheezing. Heart sounds were clear and rhythmic. Heart rate was 66 beats per minute. Сardiovascular evaluation by echocardiography revealed mitral valve prolapse and diffuse changes in myocardium. Aortic root diameter was 27 mm (normal range 18.8–25.4 mm). Abdomen was soft and painless. Ophthalmologic examination demonstrated exotropia and myopic astigmatism of both eyes. Stool was with a tendency to constipation. Micturition was normal. Intelligence was normal.

The patient had the following concomitant diseases: endemic goiter, chronic cholecystitis in remission, chronic pyelonephritis in remission, chronic pancreatitis in remission. She was on treatment with Levothyroxine sodium (150 mg/day) to treat goiter.

Differential diagnosis was carried out with progeria. Level of glucose (4.7 mmol/L) and cholesterol (5.7 mmol/L) in biochemical analysis of blood was normal, so cutis laxa was suggested.

### Patient 2 son

Height and weight at birth were 53 cm (94.5 percentile) and 3850 g (91.9 percentile), respectively. Skin laxity was first noticed at the age of 1 year.

At the age of 11 years his height and weight were 159 cm (98.9 percentile) and 34 kg (38.2 percentile) respectively. Height was above average in contrast to a small deficit in weight. The patient had thymomegaly and allergic reactions in early childhood. Сardiovascular evaluation by electrocardiography revealed single extrasystoles. Aortic root diameter was 23 mm (normal range 18,8–25,4 mm). Ophthalmologic examination demonstrated myopia of mild degree of both eyes. Intelligence was normal.

Clinical exome sequencing was done by Genotek Ltd. Ethics committee of Genotek Ltd. has approved the study (07/2018). The proband (mother) gave written informed consent to studies and publication of clinical information, images and sequencing data of her and her son.

Genomic DNA from peripheral blood samples of the proband and her son was collected using QIAamp DNA Mini Kit (Qiagen, Hilden, Germany). We did not test DNA from maternal grandparents due to the unavailability of the material. DNA libraries were constructed using the NEBNext Ultra DNA Library Prep Kit for Illumina (New England Biolabs, Ipswich, MA, USA) with adapters for sequencing on Illumina platform according to manufacturer’s protocol. For target enrichment, we used SureSelect XT2 (Agilent Technologies, Santa Clara, CA, USA). Enriched samples were sequenced using an Illumina HiSeq 2500 system (Illumina, San Diego, CA, USA) in paired-end mode (100 bp reads). We trimmed 3′-ends with read quality below 10 using Cutadapt [13]. We used SAMtools rmdup for duplicate removal [14] and FASTQS for quality control of data [15]. Sequences were analyzed as recommended by GATK Best Practices for DNA-seq [16, 17]: mapping was performed using BWA-MEM [18], variant calling using GATK HaplotypeCaller [19]. The effect of each mutation was assessed using snpEff [20]. To evaluate pathogenicity and conservation, the data was extracted from the dbNSFP [21], Clinvar [22, 23], OMIM [24] and HGMD [25]. Pathogenicity prediction by SIFT and PolyPhen-2 was not carried out due to the limitation of their use for this type of mutation. Allele frequency information was obtained from 1000 Genomes project [26, 27], ExAC [28, 29] and Genotek frequency data. Pathogenicity was predicted according to the Standards and Guidelines developed by ACMG (American College of Medical Genetics and Genomics), AMP (Association for Molecular Pathology) and CAP (College of American Pathologists) [30]. Copy number alterations were determined using CNVkit [31].

Using Sanger sequencing, the new identified *ELN* pathogenic mutation was confirmed in the proband and her son.

## Discussion and conclusions

**Fig. 2:**
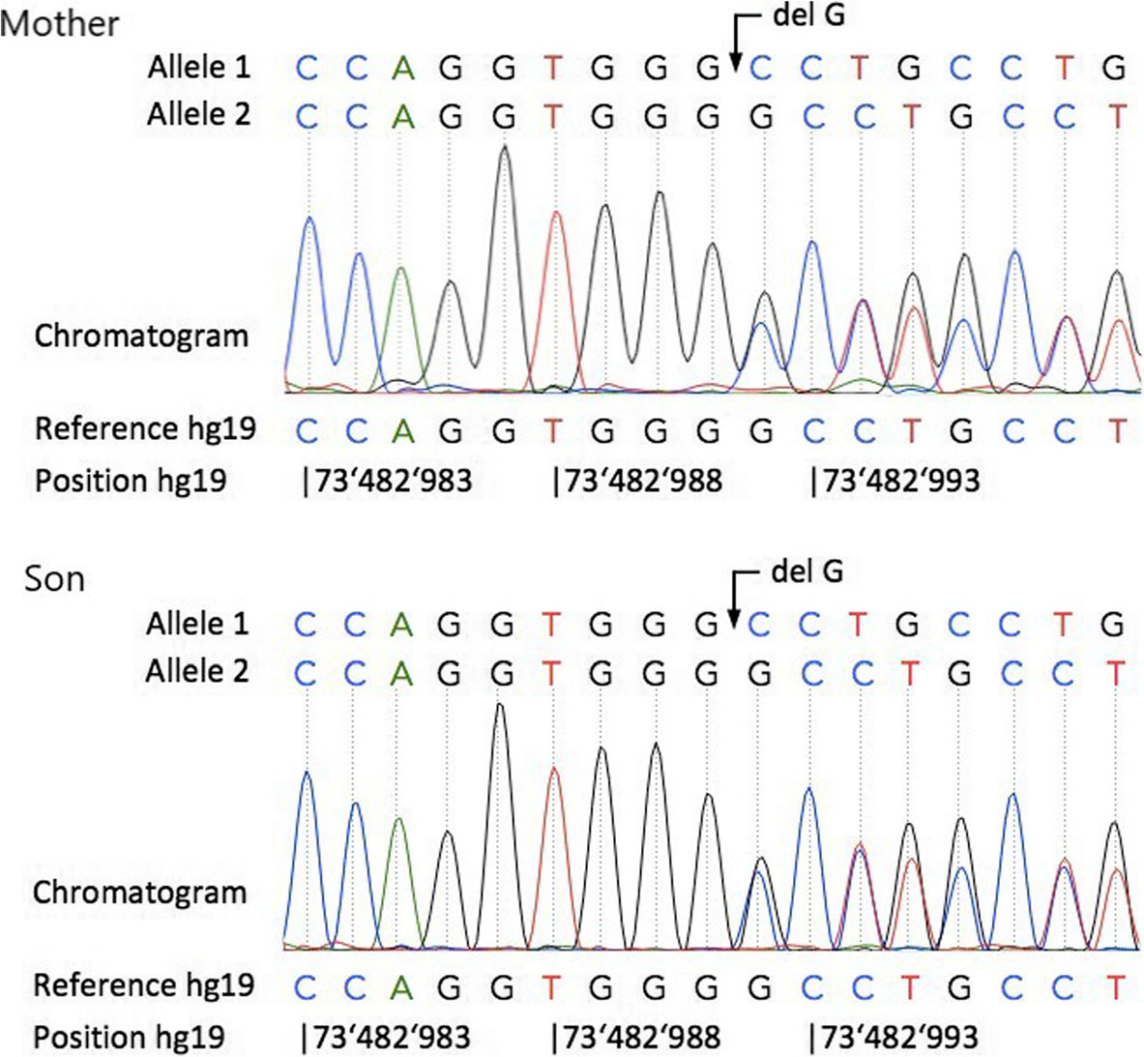
DNA sequencing chromatograms from proband (Mother) and her son (Son) showing the heterozygous c.2323delG mutation in both patients

Exome analysis showed a novel heterozygous c.2323delG mutation of elastin gene in both patients (mother and son). This mutation is single nucleotide deletion in 34 exon (NM_001278939.1) and results in a shift of reading frame. Mutation was confirmed by Sаnger sequencing (Fig. 2).

This mutation was not previously reported in literature, nor in the 60,706 subjects in ExAC, the 2535 subjects in the 1000 Genomes browser, or the 2000 Genotek patients. According to ACMG recommendations, this mutation is considered to be pathogenic - deletion with frameshift.

We also found that the proband and his son have two missense mutations: the c.2738C > T (p.Ala913Val) in the *KCNH2* gene and the c.233C > T (p.Thr78Met) in *CAV3* gene. According to ACMG recommendations, both mutations are considered to be pathogenic. Both mutations were described by Vatta et al. in patient with digenic form of congenital long-QT syndrome [32].

No other pathogenic variants and CNVs were found.

We discovered a novel frameshift mutation (c.2323delG, p.Ala775fs) in a two-patient Russian family with cutis laxa, presenting with phenotypes consistent with those previously reported. Although novel, this mutation is similar to frameshift mutations previously reported in *ELN*, for example c.2156delG described by Duz et al. [33]. Frameshift mutations of human elastin gene have been identified in several families with autosomal dominant cutis laxa [5, 7]. These mutation are deletions of guanine or cytosine in distal *ELN* exons. The location of the mutation correlates with the phenotype of patients. Mutations in the last exons cause fewer cardiovascular and pulmonary abnormalities than mutations elsewhere [6, 8].

Elastin is the primary protein determinant of connective tissue elasticity and also plays a critical role in development of cardiovascular and respiratory systems [34, 35]. Therefore, these systems are often affected in cutis laxa patients. This is especially characteristic of the autosomal recessive form, however, inguinal hernias, emphysema, aortic aneurysmal disease and root dilatation has also been described in the autosomal dominant form of cutis laxa [8, 36, 37]. Some *ELN* mutations (as partial tandem duplication in the elastin locus) can lead to fatal pulmonary complications [38]. Translocations, gross deletions and point mutations that disrupt the elastin gene lead to supravalvular aortic stenosis (OMIM 185500) [39]. Our patients did not have serious failures of internal organs consistent with a typical form of ADCL. Patient described by Duz et al. [33] also had only skin findings without cardiovascular and pulmonary complications. It is likely that mutations resulting in an elongated protein lead to this clinical phenotype.

Little is known about molecular mechanisms of ADCL. Callewaert with colleagues concluded that this form of cutis laxa is associated with increased TGFβ signaling and mutation-specific differences in endoplasmic reticulum stress and apoptosis [6].

There is overlapping of clinical features of several syndromes, including progeria, congenital cutis laxa, wrinkly skin syndrome and gerodermia osteodysplastica. All these conditions are challenging for clinicians. Our case confirmed that molecular diagnosis is the only way to resolve these phenotypically similar conditions.

## Abbreviations

ACMG: American College of Medical Genetics and Genomics
ADCL: autosomal dominant cutis laxa
AMP: Association for Molecular Pathology
CAP: College of American Pathologists
CL: Cutis laxa
